# A novel approach for spot boosting selection and treatment plan optimization to enhance prompt gamma monitoring of proton therapy^☆^

**DOI:** 10.1016/j.phro.2026.101063

**Published:** 2026-08-24

**Authors:** Beatrice Foglia, Junjie Yang, Louna Chinaud, Albin Fredriksson, Rasmus Nilsson, Nicolas Depauw, Thomas Bortfeld, Guillaume Janssens, Marco Pinto, Katia Parodi

**Affiliations:** a Department for Medical Physics, Ludwig-Maximilians-Universität (LMU) München, Am Coulombwall 1, Garching, 85748, Germany; b Physics Department, École Normale Supérieure and PSL University, 24 Rue Lhomond, Paris, 75005, France; c RaySearch Laboratories AB, Eugeniavägen 18C, Stockholm, 11368, Sweden; d Department of Radiation Oncology, Massachusetts General Hospital (MGH), 55 Fruit Street, Boston, MA, 02114, USA; e Ion Beam Applications (IBA) SA, 3 Chemin du Cyclotron, Louvain-la-Neuve, 1348, Belgium

**Keywords:** Proton therapy, Adaptive therapy, Range monitoring, Boosting, Treatment planning, Prompt gamma

## Abstract

**Background and Purpose:**

Despite continuous progress, proton therapy remains challenged by delivery uncertainties. *In vivo* monitoring can help address these limitations. Prompt-gamma (PG) imaging is a monitoring technique suitable for adaptive particle therapy, though it suffers from low signal at typical fraction doses. A treatment planning strategy was evaluated, that boosts selected spots to enhance monitoring.

**Material and Methods:**

A prostate and a head-and-neck plans were generated with a research version of RayStation (RaySearch, Sweden) treatment planning system. A detailed workflow was implemented to select candidate spots to artificially increase the number of protons (boosting) delivered according to specific criteria. Two boosting modalities were applied: (1) assigning a fixed number of protons per boosted spot; (2) maximizing the number of protons, given a lower bound. Reoptimized, boosted plans were compared to the initial one in terms of dose, dose-averaged linear-energy-transfer (LET), LET-weighted dose and robustness.

**Results:**

Boosted plans preserved target coverage and normal tissue sparing until a boost level of around 7 × 10^8^ protons, although dependent on patient case and number of boosted spots. Tumor coverage was ensured after dose rescaling. Sparing of normal tissue was deemed acceptable compared to the initial plan, until a certain boost level. *In silico* PG signals after boosting confirmed higher number of counts and noise level reduction.

**Conclusions:**

Spot boosting is a feasible approach to enhance monitoring with secondary radiation, like PG, without compromising the plan quality. Its integration into clinics could enable an improved delivery quality assurance and a more precise proton therapy.

## Highlights

• Boosting selected spots in proton therapy enhanced prompt-gamma-based monitoring.
• The quality of the boosted plans was equivalent to the one of initial plans.
• Target coverage and normal tissue sparing was preserved after boosting.
• Noise in prompt-gamma profiles decreased on average up to 93.3% ± 1.7% after boosting.

## Graphical abstract

**Figure:**
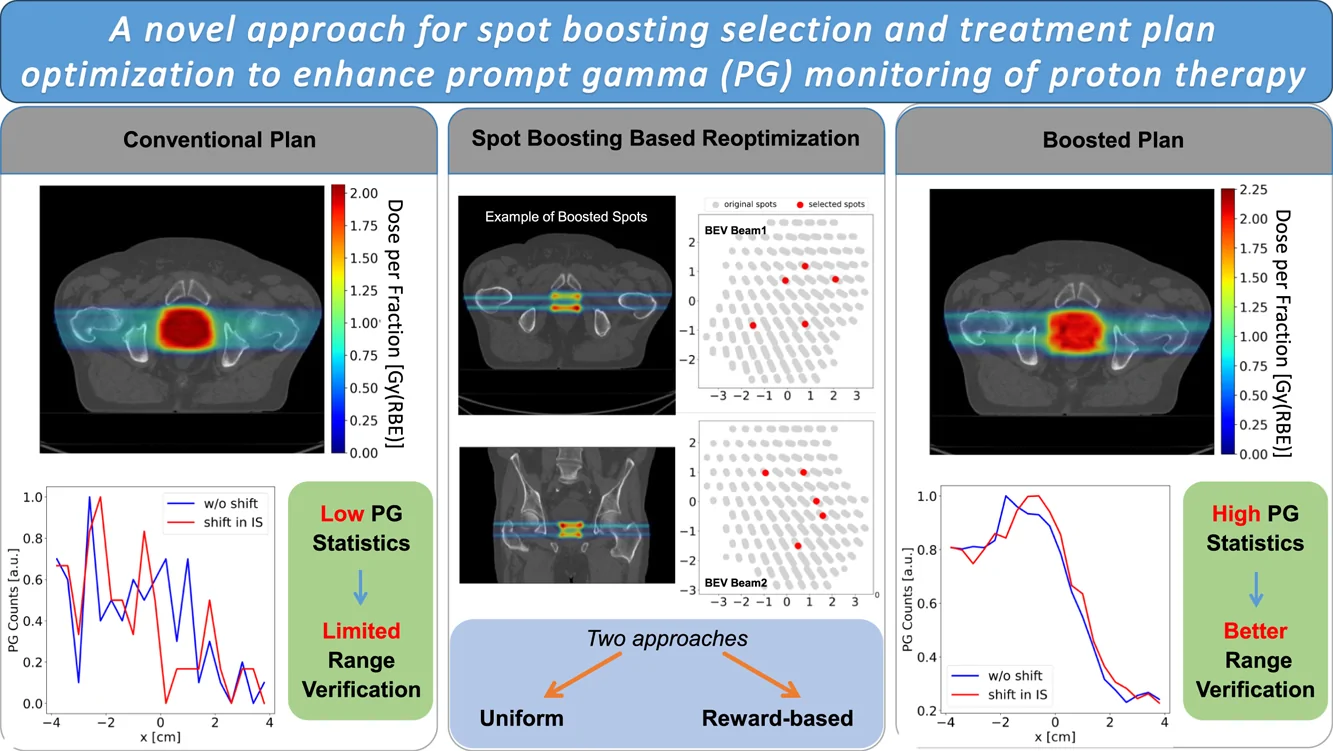

## 1 Introduction

Proton therapy ballistic advantages are limited in clinical practice by delivery uncertainties [1], [2], [3], [4] arising from X-ray computed tomography imaging, dose calculation, treatment delivery and anatomical changes, affecting proton range and dose distribution. To mitigate these uncertainties, robust optimization [5] is employed, where different treatment scenarios are considered into the intensity modulated proton therapy (IMPT) treatment optimization. Another solution is the addition of safety margins [1]. Both the use of margins and robust optimization increase dose to the surrounding tissue.

Adaptive proton therapy [4], [6], [7] offers a dynamic approach to adjust treatment plans based on feedback. While adaptive workflows exist for conventional radiotherapy, adaptive proton therapy is in early clinical trials [8], [9] and attracting interest.

Fast *in vivo* range monitoring is crucial for adaptive workflows [7]. Secondary radiation detectable outside the patient, such as positron emission tomography (PET) photons [10], prompt gammas (PG) [11], [12], or ionoacoustic signals [13], could be exploited for range monitoring [2], [14]. PG emission occurs within nanoseconds, potentially allowing real-time verification at spot level in pencil beam scanning [15], [16], but suffers from low yields [12]. PG monitoring requires more than 2 × 10^8^ protons for a range verification precision better than 2 mm for a measured PG profile using a knife-edge slit camera [15]. This is not considered in current treatment planning. Tian et al. [17] proposed a new treatment planning concept, which increases the number of protons (boosting) for a few spots selected based on indicators of dose–PG conformity, ensuring proton numbers exceed the 2 × 10^8^ desired detectability threshold. Reoptimized boosted plans exhibited a robust dose/volume metric quality comparable to the initial plan, and showed potential to reach 0.8 mm precision with *in silico* PG profiles at emission [17], [18], [19].

The aim of this work was to enhance prompt-gamma-based monitoring in proton therapy by boosting selected spots of a treatment plan. In particular, it aimed to extend the above-mentioned previous work to more clinically realistic treatment plans by using, for the first time, a research version of a modern, widely used commercial treatment planning system (TPS), and by introducing a rigorous spot selection workflow that does not require prior PG calculation.

## 2 Materials and methods

Representative treatment plans, generated for research purposes only and not intended for clinical delivery, were created for a prostate case and a head-and-neck (H&N) case using a research version of RayStation 2023B (RaySearch, Sweden). The plans assumed a ProteusONE proton therapy system (Ion Beam Applications, Belgium) and were generated under the guidance of a medical physics expert.

For all plans, a constant relative biological effectiveness (RBE) of 1.1 was assumed. For the prostate case, 40 Gy(RBE)^3^ median dose was prescribed to the gross tumor volume (GTV, coincident with the prostate) over 20 fractions. Two antiparallel left–right (LR) and right-left (RL) IMPT beams were considered. Robust optimization accounted for 5 mm setup and 3.5% density uncertainties, resulting in 11 energy layers per beam with energies between 146.5 and 182.1 MeV.

The H&N case involved a nasopharyngeal tumor. Following the literature [20], [21], three IMPT beams were used: one superior-anterior oblique beam (SAO, 20°) and two anterior-posterior oblique, left (APL, 25°) and right (APR, 25°). A dose of 69.96 Gy(RBE) was prescribed to 95% of the clinical target volume (CTV, a 5 mm expansion of the GTV). Robust optimization was applied to the CTV and relevant organs of interest (OOIs), assuming 3 mm positional and 3% density uncertainties. This resulted in 35 energy layers for the SAO beam and 31 for the APL and APR beams, with energies from 70 to 152.8 MeV. Further details are provided in Tab. S1 of the Supplementary Material.

The following workflow was implemented to identify suitable spots for boosting. Boosted spots were chosen to stop within the tumor volume (step 1), identified as the planning target volume (PTV) for the prostate and the CTV for the H&N case. They were distant from air cavities (step 2, as in [17]) and the tumor margin (step 3), the latter to prevent hotspots in the normal tissue under range uncertainties. Distances were determined once and applied universally by analyzing lateral dose profiles and differences along the beam direction between depth-dose and PG profiles simulated in water using Geant4 version 10.07.p03 over the therapeutic energy range [22]. Boosted spots must deliver minimum normal tissue dose relative to tumor dose (step 4), as quantified by $overlap_{spot,OOIs}=\frac{D_{tumor}}{V_{tumor}}-\sum_{i=1}^{N}\frac{D_{OOI,i}}{V_{OOI,i}}$ where N is the number of critical OOIs, D the spot dose, and V the volume. Half of the spots, those with the lowest $overlap_{spot,OOIs}$, were kept. To ensure spatial spread and avoid overlap among candidates, spots were divided in beam’s eye view (BEV) into 10 clusters using the k-means algorithm (step 5) [23]. For each cluster, the spot with the deepest path in the patient was chosen, as longer paths traverse more tissue and enhance monitoring effectiveness. Spots’ overlap was evaluated to prevent concentration of boosted spots (step 6). For each spot, the planned dose D was normalized by proton number. For each pair of spots i and j delivering dose to a voxel k, a correlation value was calculated as $overlap_{i,j}=\frac{\sum_{k=1}^{M}D_{ik}D_{jk}}{\sqrt{\sum_{k=1}^{M}D_{ik}^{2}\cdot \sum_{k=1}^{M}D_{jk}^{2}}}$ where M is the total number of voxels in the dose grid. Each spot’s final value corresponded to the sum of its correlations with all the candidate spots across all beams, including its own.

**Fig. 1:**
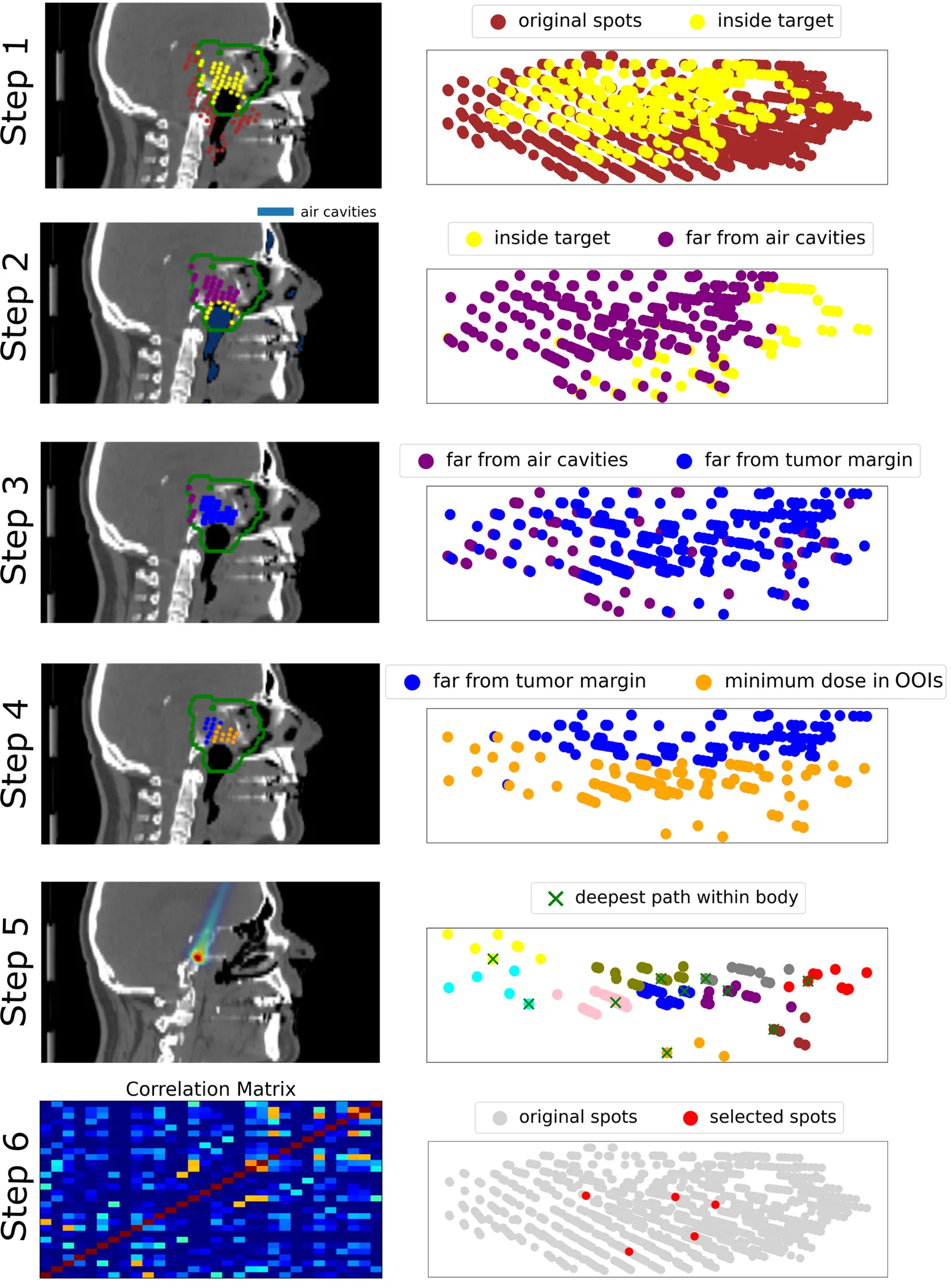
Representative scheme of the workflow to select the spots to be boosted. The SAO beam for the H&N patient is shown as an example. The first column shows the sagittal view of the CT. In the second column, spots in BEV are reported at every step. The CTV contour is highlighted in green from step 1 to step 4. In step 5 CT column, a single spot dose is showed as an example. Voxels identified by the workflow as air cavities are highlighted in dark blue in step 2 CT. The different colors in step 5 BEV are meant to highlight the different clusters.

Spots were ranked by overlap degree, and selected in order. After selecting from 1 to 5 spots per beam, the plan was reoptimized. Fig. 1 shows how the number of candidate spots evolved after each workflow step. To validate the workflow, additional plans were generated with random spot selection after step 3, creating 30 configurations reoptimized under the same conditions, or by excluding single workflow steps.

The research version of RayStation used herein was extended with a new functionality allowing, during optimization, a custom bound on the number of protons delivered to selected spots. The number of protons initially (i.e., before boosting) assigned by the TPS optimizer to candidate spots and other spot information are shown in Table 1. Defining a spot’s weight by the number of protons is less common than using monitor units (MU) in clinical practice, but conversions are straightforward via facility calibration data defining the number of protons per MU depending on energy.

Two boosting modalities were considered. In the first, each candidate spot received the same number of protons (2 × 10^8^ – 8 × 10^8^), increasing its weight by one to two orders of magnitude compared to the initial plan. The second modality introduced a “reward” objective function: a lower bound was set for boosted spots, and the TPS optimizer aimed to increase the number of protons as much as possible while balancing other objectives. The reward function is

(1) $$f\left(NP_{1},\ldots ,NP_{N}\right)=w\cdot \overset{N}{\underset{i=1}{\sum }}e^{-s\cdot \left(NP_{i}-NP_{LB}\right)}$$

where N is the number of boosted spots, NP the number of protons, and LB the lower bound. The negative exponent makes the reward function exponentially smaller as $NP_{i}$ increases. $NP_{LB}$ was fixed at 2 × 10^8^ as the threshold mentioned in Section 1. Weight w and steepness s were user-defined. After 20 optimization iterations for prostate and 100 for H&N, spots with weight below 0.02 MU per fraction, corresponding to 1.5 × 10^6^ – 3 × 10^6^ protons depending on incident energy, were removed.

**Table 1:** Properties of the spots selected for boosting: energy, number of protons initially assigned by the optimizer, and spot size at the exit of the nozzle (full width at half maximum, FWHM). The spot size values are reported until the first changing digit.

Patient	Beam	Boosted	Energy	Initial number	Spot size
	field	spot ID	(MeV)	of protons (×10^6^)	(FWHM, cm)
Prostate	LR	0	163.8	2.8	0.93
		1	174.4	81.7	0.87
		2	171.0	23.7	0.89
		3	171.0	2.8	0.89
		4	171.0	16.1	0.89
	RL	0	171.2	31.3	0.88
		1	167.7	3.3	0.90
		2	167.7	2.9	0.90
		3	171.2	12.0	0.88
		4	167.7	2.8	0.90

H&N	SAO	0	113.4	38.3	1.25
		1	133.3	4.9	1.13
		2	88.1	8.7	1.51
		3	133.3	27.3	1.13
		4	115.9	2.1	1.23
	APL	0	76.1	1.6	1.68
		1	98.0	1.9	1.39
		2	87.3	12.5	1.51
		3	89.4	1.8	1.49
		4	114.9	2.8	1.24
	APR	0	97.5	5.8	1.39
		1	93.2	22.8	1.44
		2	117.0	24.2	1.22
		3	111.9	2.1	1.26
		4	109.5	2.2	1.28

Boosted plans were compared to the initial one in terms of RBE-weighted dose (D), dose-averaged linear-energy-transfer (LET) ($LET_{D}$), LET-weighted dose ($D_{LET}$) distributions, and related volume histograms DVH, LVH, and LDVH. $D_{LET}$ [24] is defined as

(2) $$D_{LET}=D_{PHYS}\times \left(1+\kappa LET_{D}\right)$$

where $D_{PHYS}$ is the absorbed dose, and κ is an empirical fitting parameter with units of μm/keV. A value of 0.055μm/keV was used, as suggested by [24].

Boosted plan quality was evaluated using D${}_{98\text{\%}}$, D${}_{2\text{\%}}$, L${}_{98\text{\%}}$, L${}_{2\text{\%}}$, LD${}_{98\text{\%}}$, and LD${}_{2\text{\%}}$. Relative differences RD between boosted and initial plan metrics were also calculated. To ensure target coverage, boosted plans were rescaled so that tumor nominal D${}_{98\text{\%}}$ matched the initial plan.

Plan robustness was also evaluated using RayStation functionalities, considering 3% density and 3 mm position uncertainties for the H&N case, and 3.5% density and 5 mm position uncertainties for the prostate case. For all 16 scenarios obtained with these settings for both patients, target D${}_{98\text{\%}}$ and the D${}_{2\text{\%}}$ of OOIs were collected, and absolute differences with the initial plan scenarios were calculated.

PG distributions from boosted spots were retrieved *in silico* using the open-source MATLAB toolbox REGGUI (Ion Beam Applications SA, Belgium) to evaluate the benefit of higher statistics of primary protons for monitoring. A realistic PG signal of a knife-edge slit camera [25], consisting of a LYSO crystals-based photodetection system beyond a knife-edge slit tungsten collimator, was considered. The analytical knife-edge slit camera response [26] was simulated with REGGUI for the typically considered energy window of 3 – 6 MeV. Since the analytical model in REGGUI does not account for primary count, Poisson noise was added to simulate statistical fluctuations. Noise was applied 100 times, and the statistical uncertainty was calculated as one standard deviation (1 σ).

**Fig. 2:**
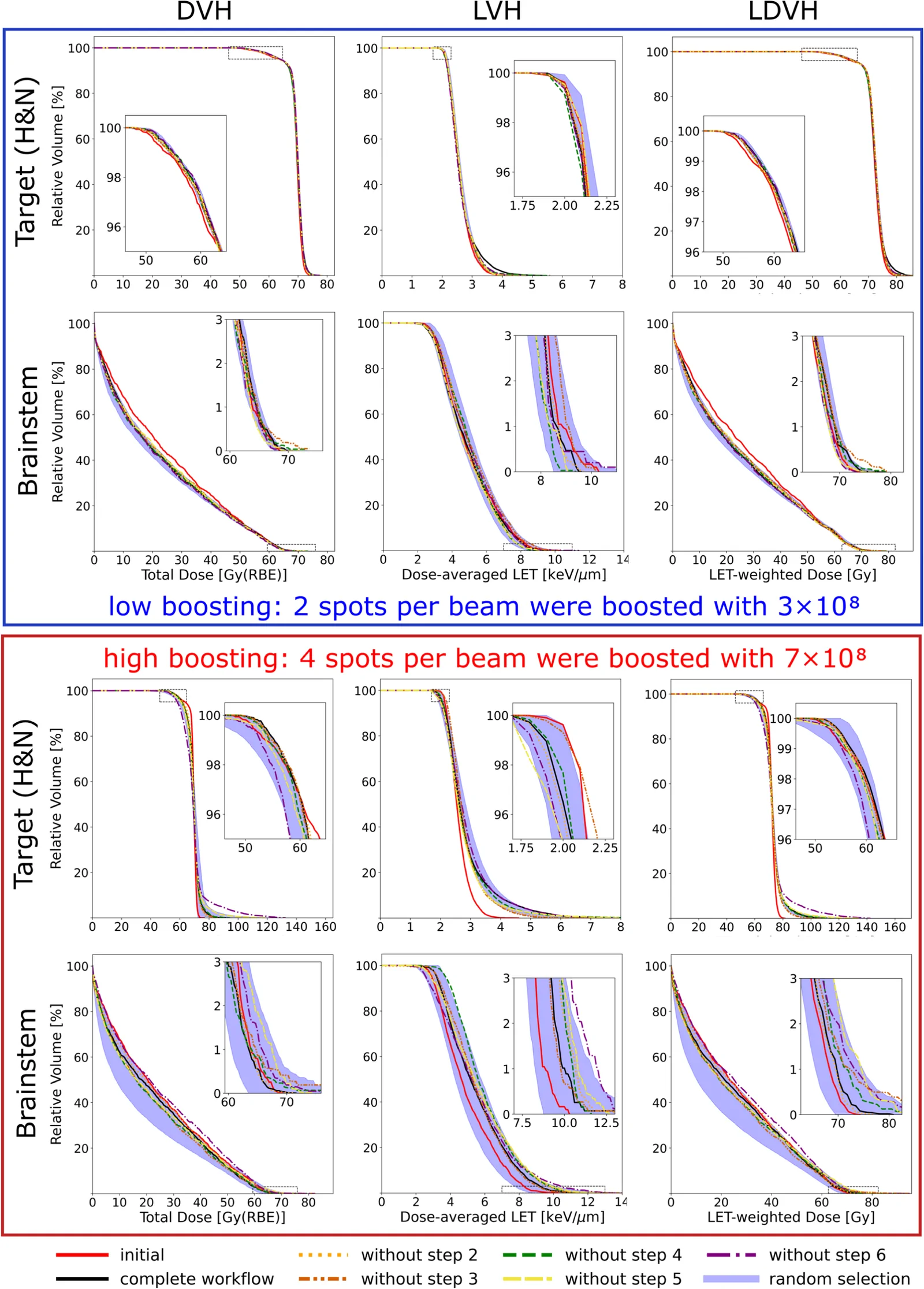
DVHs (first column), LVHs (second column) and LDVHs (third column) of a low (blue box) and a high (red box) boosting scenario for the H&N case. First and third row: H&N target volume, zoomed in the 98% volume region. Second and fourth row: brainstem, chosen as an exemplary OOI and zoomed in the 2% volume region. The blue band results from the workflow with the random selection of spots to be boosted, and illustrates the distribution of the volume-histograms for 30 configurations, within 2 σ. D${}_{98\text{\%}}$ rescaling was not applied.

**Table 2:** D${}_{2\text{\%}}$ (Gy(RBE)), L${}_{2\text{\%}}$ (keV/μm) and LD${}_{2\text{\%}}$ (Gy) for the brainstem contour for all the boosting cases evaluated, including D${}_{98\text{\%}}$ scaling, and relative differences (in brackets) to the plan before boosting, exhibiting initial values of D${}_{2\text{\%}}=62.3\;\mathrm{Gy}$(RBE), L${}_{2\text{\%}}=8.4\;\mathrm{keV}$/μm, LD${}_{2\text{\%}}=66.4\;\mathrm{Gy}$. Plans were boosted with the same number of protons per selected spot.

# Protons per	# Boosted spots per beam				
Boosted spot (×10^8^)					
D${}_{2\text{\%}}$	1	2	3	4	5
2	62.0 (−0.5%)	62.1 (−0.3%)	61.7 (−0.9%)	61.7 (−0.9%)	61.2 (−1.8%)
3	61.5 (−1.3%)	62.0 (−0.5%)	61.6 (−1.1%)	61.4 (−1.4%)	61.3 (−1.7%)
4	61.7 (−1.0%)	61.2 (−1.8%)	61.7 (−1.0%)	62.0 (−0.4%)	61.3 (−1.7%)
5	62.0 (−0.6%)	61.0 (−2.1%)	61.7 (−1.0%)	61.0 (−2.1%)	60.6 (−2.7%)
6	61.6 (−1.1%)	61.7 (−1.0%)	60.8 (−2.4%)	62.2 (−0.2%)	61.0 (−2.1%)
7	61.5 (−1.4%)	62.2 (−0.2%)	62.1 (−0.4%)	62.2 (−0.2%)	61.8 (−0.8%)
8	61.9 (−0.7%)	63.1 (1.2 %)	63.2 (1.4%)	62.3 (0.0%)	63.5 (1.9%)

L${}_{2\text{\%}}$	1	2	3	4	5

2	8.2 (−3.4%)	8.3 (−2.1%)	8.5 (0.5%)	8.4 (−1.1%)	8.4 (0.0%)
3	7.9 (−6.2%)	8.3 (−1.7%)	8.4 (−1.0%)	8.3 (−1.5%)	8.6 (1.3%)
4	8.2 (−3.5%)	8.5 (1.1%)	8.7 (3.4%)	8.5 (1.1%)	8.9 (5.5%)
5	8.5 (0.1%)	8.6 (1.8%)	9.0 (6.3%)	9.3 (10.1%)	8.9 (5.9%)
6	8.7 (2.7%)	8.7 (2.9%)	9.0 (6.1%)	9.5 (12.8%)	9.1 (7.4%)
7	8.3 (−1.5%)	8.5 (0.1%)	9.1 (7.2%)	9.4 (11.8%)	9.5 (12.4%)
8	8.6 (1.5%)	9.1 (8.3%)	9.2 (8.4%)	10.2 (20.7%)	9.8 (16.0%)

LD${}_{2\text{\%}}$	1	2	3	4	5

2	65.9 (−0.8%)	66.3 (−0.2%)	65.7 (−1.1%)	66.4 (−0.1%)	65.5 (−1.4%)
3	65.8 (−1.0%)	66.2 (−0.3%)	66.3 (−0.2%)	66.1 (−0.5%)	65.8 (−0.9%)
4	66.2 (−0.3%)	65.7 (−1.1%)	66.7 (0.4%)	67.2 (1.3%)	66.1 (−0.4%)
5	66.2 (−0.3%)	65.7 (−1.1%)	66.5 (0.2%)	66.3 (−0.2%)	65.6 (−1.2%)
6	65.9 (−0.9%)	66.3 (−0.2%)	66.6 (0.2%)	67.7 (2.0%)	66.5 (0.1%)
7	65.5 (−1.4%)	66.8 (0.6%)	67.3 (1.3%)	67.8 (2.1%)	67.9 (2.2%)
8	66.3 (−0.3%)	68.0 (2.4%)	69.1 (4.1%)	68.9 (3.7%)	69.4 (4.5%)

## 3 Results

The effects of the spot selection workflow steps are shown in Fig. 2 for both low and high boosting scenarios in the H&N case. At low boosting, the steps of the spot selection workflow minimally affected target coverage and normal tissue sparing. On average, CTV D${}_{98\text{\%}}$ changed by ＋1.3% ± 0.6%, L${}_{98\text{\%}}$ by −1.4% ± 0.9%, and LD${}_{98\text{\%}}$ by ＋1.0% ± 0.4%, while brainstem D${}_{2\text{\%}}$, L${}_{2\text{\%}}$, and LD${}_{2\text{\%}}$ changed by ＋0.6% ± 0.5%, −2.0% ± 3.1%, and ＋0.5% ± 0.7%, respectively. At higher boosting, CTV D${}_{98\text{\%}}$, L${}_{98\text{\%}}$, and LD${}_{98\text{\%}}$ changed by −0.7% ± 1.9%, −7.3% ± 4.1%, and −0.7% ± 1.2%, while brainstem D${}_{2\text{\%}}$, L${}_{2\text{\%}}$, and LD${}_{2\text{\%}}$ increased by 0.3% ± 1.8%, 19.0% ± 6.7%, and 3.3% ± 2.1%. Omitting the clustering step had the effect of changing brainstem D${}_{2\text{\%}}$, L${}_{2\text{\%}}$ and LD${}_{2\text{\%}}$ by −3.5%, 22.4% and 6.5%, respectively, while omitting the step evaluating the spot–organ overlap changed them by -1.8%, 19.1% and 3.2%, respectively. Lower CTV D${}_{98\text{\%}}$ indicated insufficient target coverage. Therefore, D${}_{98\text{\%}}$ rescaling was applied, altering the number of protons assigned to boosted spots, on average by between 1.4% ± 0.1% and 6.0% ± 2.6% for prostate, while for H&N they ranged from −2.0% ± 0.2% to 1.2% ± 1.1% (Table S2 of the Supplementary Material).

OOI analysis on the brainstem (Table 2) showed that selecting 1 to 5 spots per beam with the complete workflow produced maximum D${}_{2\text{\%}}$, L${}_{2\text{\%}}$ and LD${}_{2\text{\%}}$ differences of 3%, 21% and 5%, respectively. Same analysis on the rectum showed maximum differences of 11%, 15% and 12%, respectively (Tab. S3 of the Supplementary Material).

**Fig. 3:**
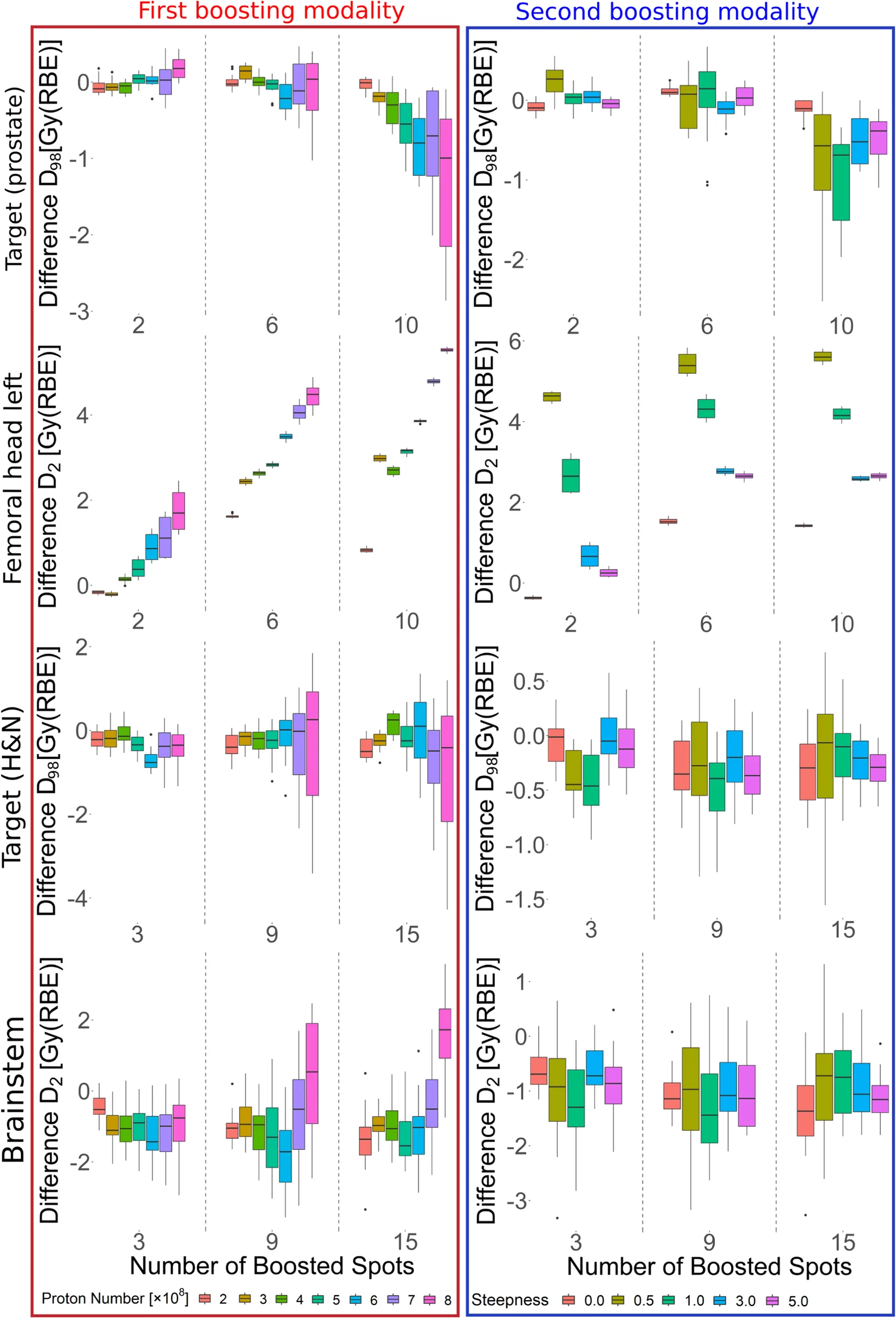
Robustness evaluation of the boosted plans and comparison with the initial one, reporting the absolute differences in the metrics between respective scenarios for the target volume and one selected OOI for the prostate (first row) and H&N (second row). Both boosting modalities are here shown. Only the cases of 1, 3 and 5 boosted spots per beam are reported. Please note that the plot scales vary.

With the reward function, optimizer-assigned boosted protons ranged from (2.25 ± 0.26) × 10^8^ protons to (6.02 ± 0.44) × 10^8^ for H&N and from (3.31 ± 0.09) × 10^8^ to (7.64 ± 0.21) × 10^8^ for prostate. The reward function reduced the maximum D${}_{2\text{\%}}$, L${}_{2\text{\%}}$ and LD${}_{2\text{\%}}$ differences to about 2%, 15% and 2%, respectively for the brainstem. The results for prostate were closer between the two modalities (Tab. S4–S5 of the Supplementary Material). Target coverage robustness decreased with higher boosting (Fig. 3: the D${}_{98\text{\%}}$ difference with the initial plan reached a standard deviation value of 1.1 Gy(RBE) for prostate and 1.9 Gy(RBE) for H&N for the first boosting modality, while for the second boosting modality these values were up to 0.9 Gy(RBE) for prostate and 0.7 Gy(RBE) for H&N, respectively. D${}_{2\text{\%}}$ of OOIs (Fig. 3, Fig. S2-S5 of the Supplementary Material) nominally increased, as D${}_{2\text{\%}}$ differences reached even 5.5 Gy(RBE) for the femoral head left, yet remained largely stable.

**Fig. 4:**
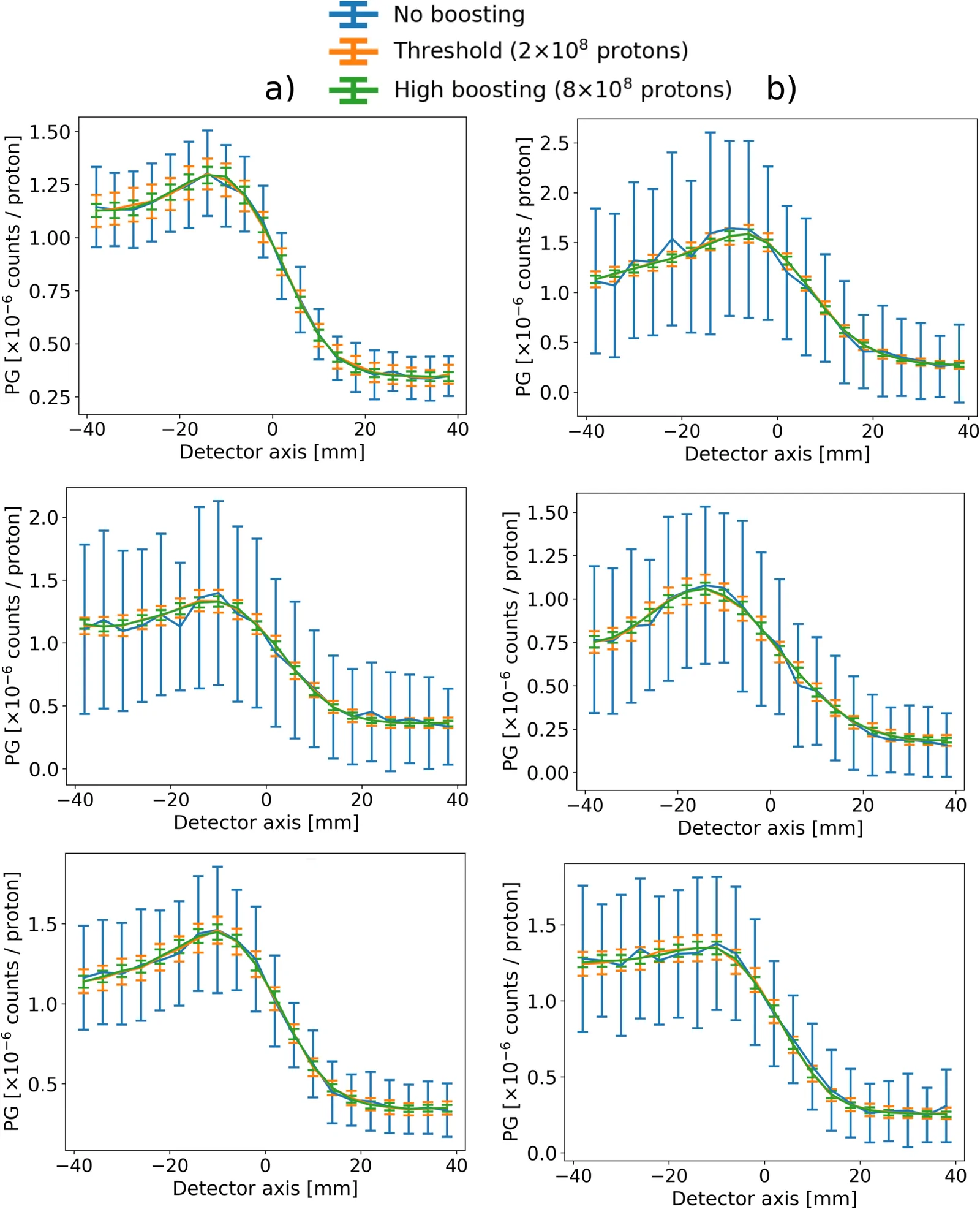
Prompt gamma signals obtained considering different numbers of incident protons for three exemplary spots of the (a) prostate and (b) H&N plans: the one of the initial plan, the detectability threshold of 2 × 10^8^ protons, and 8 × 10^8^ protons as a high boosting example. Poisson noise was introduced 100 times, and the average signal with 1σ statistical uncertainty is reported.

The noise in the *in silico* PG profiles at detection level for three spots in both prostate and H&N cases decreased of 76.6% ± 9.7% for the prostate and 86.6% ± 3.5% for the H&N when increasing the number of incident protons from the initial value to the threshold of $2\times 10^{8}$, and of 88.1% ± 4.9% for the prostate and 93.3% ± 1.7% for the H&N when reaching $8\times 10^{8}$ protons (Fig. 4).

## 4 Discussion

This work confirmed that it is possible to reoptimize IMPT plans enhancing *in vivo* monitoring capabilities via boosting selected spots without necessarily compromising the dose/volume metric quality, even when the number of protons per boosted spot exceeds the proposed PG detectability threshold of 2 × 10^8^ [15]. This was achieved using a research version of a state-of-the-art TPS, which could support the clinical translation of the approach described in this manuscript.

Boosting in the treatment planning phase with the goal of improving *in vivo* monitoring may be counter-intuitive at first glance. In fact, monitoring is usually employed with the purpose of verifying and possibly adapting the treatment plan, rather than the other way around [10], [27]. But both are intrinsically connected: boosting at the planning stage enhances the monitoring performance to in turn enable a better plan adaptation.

A novel spot selection workflow independent of the PG prediction was central to the methodology. Not only was the spatial location of candidate spots within the tumor considered, but also their relative dose to OOIs and overlap, to avoid generating unwanted hotspots.

Different levels of boosting were tested, with 1 to 5 spots per field and an increasing number of protons per spot from 2 × 10^8^ to 8 × 10^8^ in the fixed boosting scheme. In case multiple pulses might be needed to deliver the assigned number of boosted protons because of machine limitations, PG signals from the entire delivery of those spots could be merged. The boosting range was intentionally pushed to extreme values to determine the threshold beyond which plan quality begins to deteriorate, observed in this study at around 7 × 10^8^ protons, although dependent on the patient case and on the number of boosted spots.

To optimize the degree of boosting while balancing plan quality and PG detectability, a “reward” function was introduced that set a lower bound on the number of protons per boosted spot and allows the optimizer to assign the maximum feasible number of protons while still fulfilling planning objectives. The reward function was modeled as a decreasing exponential. Other formulations could be explored, depending on the optimization goals or based on computational considerations.

Alternative boosting modalities have been studied. The first one controlled the amount of boost, since the number of protons is assigned directly by the user. In the “reward” scenario, the degree of boosting beyond a threshold was left to the optimizer, to balance proton spot statistics and plan quality. A reasonable boost was found for relatively low values of steepness. Higher steepness values pushed the “reward” objective function too fast towards the minimum, and the assigned number of protons was very close to the set lower bound. Higher weights instead pushed the boost too far, and the plan quality was degraded.

Boosted plans were evaluated not only in terms of dose distributions, as in [17], [18], [19], but also $LET_{d}$ and $D_{LET}$, providing insight into potential radiobiological effects. A κ value of 0.055μm/keV was used for $D_{LET}$, consistent with previous studies [24]. This value is highly site-dependent and should ideally be validated through radiobiological experiments or clinical outcome. Therefore, comparisons focused on relative rather than absolute metrics.

The reoptimization approach yielded better outcomes for the H&N case compared to prostate, particularly for higher boosting levels. This was attributed to the tumor volume: larger target volumes, such as the 118 cm^3^ H&N target versus the 29 cm^3^ prostate target, offer more candidate spots for boosting, which facilitates maintaining plan quality.

Plan acceptability was assessed through D${}_{98\text{\%}}$ for the tumor and D${}_{2\text{\%}}$ for OOIs. To guarantee target coverage, plans were rescaled after optimization so that D${}_{98\text{\%}}$ matched that of the initial plan. This rescaling slightly altered the actual number of protons per spot, but the effect was minimal, representing an acceptable trade-off between boosting and preserving the prescription of the radiation oncologist. Changes in OOIs metrics compared to the initial plan were generally within the original prescription limits, described as objective functions in Tab. S1 of the Supplementary Material. Objective function weights could be adjusted if stricter constraints are required.

Previous work by Van de Water et al. [28], [29] demonstrated that iterative spot optimization, adding randomly selected spots and excluding low-weight spots until plan quality deteriorates, could substantially reduce IMPT delivery time while maintaining plan quality. The workflow used in this study similarly reduced the number of spots and, therefore, the potential treatment time, particularly when higher boosting is applied, although this effect was not quantitatively evaluated here. Our focus was not only on reducing low-weight spots but also on ensuring that boosted spots avoid overlaps that could create hotspots or compromise the reliability of PG monitoring. The reported results indicated that the increased number of protons assigned to boosted spots improves PG counting statistics. In the context of adaptive radiotherapy [4], [7], improved PG signal quality may facilitate the detection of range deviations across treatment fractions and support decision-making for replanning. However, the usefulness of PG signals for range monitoring also depends on the spatial location of the selected spots. PG-based monitoring remains particularly attractive for online or adaptive verification due to its high temporal resolution [12]. In an ideal adaptive workflow, the boosted spots could be delivered at the beginning, then the rest of the treatment could be delivered or adapt after monitoring few selected areas. Candidate spots for boosting can be selected by combining the workflow described here with dose-PG correlation criteria established in prior studies [17], [18], [19], or other user-dependent choices complementary to these studies, e.g. considering boosting in regions which are considered more critical from a planning point of view.

This study was based on two representative patient cases only, involving two different anatomical sites. In both cases the applicability and benefits of spot boosting for enhancing the PG signal were demonstrated. Nevertheless, this still represents a limitation, and more patients and anatomical regions are planned to be included in future studies.

The PG signals were based on a simulated detection framework, experimentally validated in previous works [26], [30], [31]. Nonetheless, future work would benefit from considering actual detected PG data, with or without boosting.

In conclusion, it is feasible to optimize treatment plans with boosted spots, enhancing PG-based range monitoring in proton therapy and addressing the problem of low PG signal-to-noise ratio for clinical translation of *in vivo* monitoring. This was demonstrated through the evaluation of dose/volume, LET-related, and robustness metrics in prostate and H&N cases, focusing on clinical feasibility and implementation within a commercially relevant TPS. Even though the focus was on PG, it can be extended to all monitoring methods which benefit from higher statistics to enable an initial reliable verification of selected spots prior to entire delivery.

## CRediT authorship contribution statement

**Beatrice Foglia:** Writing – original draft, Visualization, Validation, Software, Methodology, Investigation, Data curation. **Junjie Yang:** Writing – original draft, Visualization, Validation, Software, Investigation, Formal analysis, Data curation. **Louna Chinaud:** Writing – review & editing, Validation, Investigation, Data curation. **Albin Fredriksson:** Writing – review & editing, Supervision, Software, Resources, Project administration, Methodology, Data curation. **Rasmus Nilsson:** Writing – review & editing, Supervision, Software, Resources. **Nicolas Depauw:** Writing – review & editing, Validation, Supervision. **Thomas Bortfeld:** Supervision, Resources. **Guillaume Janssens:** Writing – review & editing, Software, Resources. **Marco Pinto:** Writing – review & editing, Supervision, Resources, Methodology, Conceptualization. **Katia Parodi:** Writing – review & editing, Supervision, Resources, Project administration, Methodology, Funding acquisition, Conceptualization.

## Ethics statement

This study was conducted as a retrospective *in silico* analysis of already existing, strictly anonymized patient data for research purposes only. Data came from two centers: the H&N (nasopharingeal) case from LMU Klinikum (Munich, Germany) and the prostate case from RaySearch Laboratories AB (Stockholm, Sweden). In accordance with both German (Bavarian Hospital Law, Art. 27 Abs. 4 BayKrG) and Swedish national regulations, formal Ethics Committee approval was not required as the study relied exclusively on retrospective, de-identified data. All data were fully anonymized prior to access by the authors, ensuring no direct or indirect identification of individuals. Research was conducted in accordance with the Declaration of Helsinki and the principles of Good Scientific Practice (GSP). No clinical interventions were performed, and the results are based solely on secondary analysis collected data.

## Declaration of competing interest

The authors declare that they have no known competing financial interests or personal relationships that could have appeared to influence the work reported in this paper.

## Appendix A Supplementary data

MMC S1

## Footnotes

3 Referring to the ICRU93 definition.
