# Supplementary material for: A novel approach for spot boosting selection and treatment plan optimization to enhance prompt gamma monitoring of proton therapy
Source: Phys Imaging Radiat Oncol. 2026 Aug 24;41:101063. doi: 10.1016/j.phro.2026.101063 (PMC13545157; doi:10.1016/j.phro.2026.101063)
Supplement: Supplementary file 1 [file mmc1.pdf]

A novel approach for spot boosting selection and treatment plan optimization to enhance prompt gamma monitoring of proton therapy

Beatrice Foglia[a,*], Junjie Yang[a,*], Louna Chinaud[b], Albin Fredriksson[c], Rasmus Nilsson[c], Nicolas Depauw[e], Thomas Bortfeld[e], Guillaume Janssens[1], Marco Pinto[a], Katia Parodi[a,**]

[a]*Department for Medical Physics, Ludwig-Maximilians-Universität (LMU) München, Am Coulombwall 1, Garching, 85748, Germany*
[b]*Physics Department, École Normale Supérieure and PSL University, 24 Rue Lhomond, Paris, 75005, France*
[c]*RaySearch Laboratories AB, Eugeniavägen 18C, Stockholm, 11368, Sweden*
[d]*Department of Radiation Oncology, Massachusetts General Hospital (MGH), 55 Fruit Street, Boston, MA, 02114, USA*
[e]*Ion Beam Applications (IBA) SA, 3 Chemin du Cyclotron, Louvain-la-Neuve, 1348, Belgium*

---

[*]These authors contributed equally to this work, share first authorship, and are listed alphabetically.
[**]Corresponding author: katia.parodi@physik.uni-muenchen.de

Supplementary Material

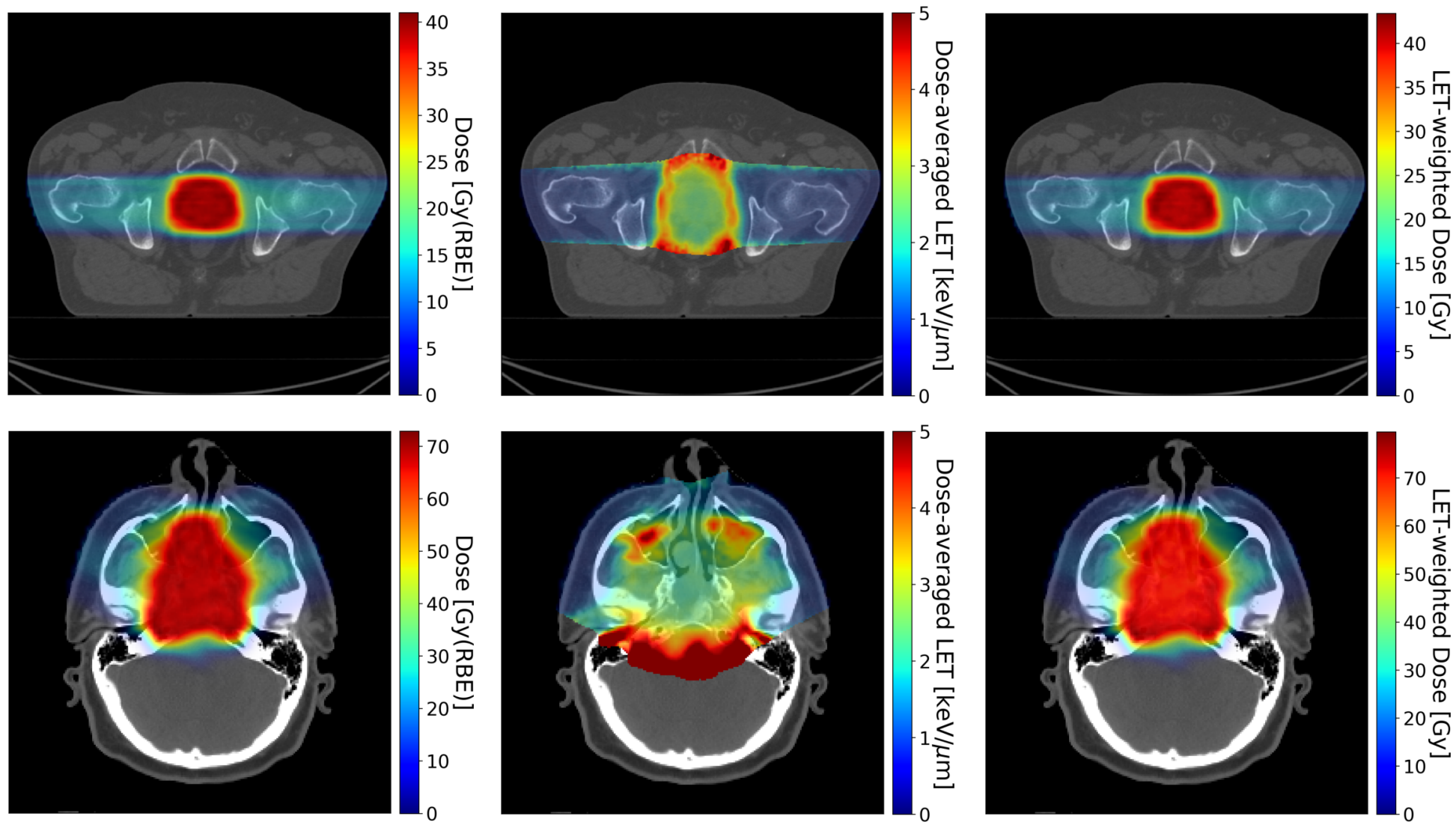

Fig. S1: Axial views of dose, dose-averaged LET and LET-weighted dose distributions for the initial plans considered in this work. First row: prostate case. Second row: H&N case. For a matter of readability, the dose-averaged LET is shown only when the dose values in the voxels exceed a value of 1 % of the maximum, and the maximum value of the LET was set to 5 keV/μm.

Table S1: Properties of considered plans. Only the objective functions considered more relevant are listed. OOI stands for "organ of interest". Gy(RBE) refers to the ICRU93 definition.

**Patient case**	**OOI**	**Objective**	**Value**	**Weight**
**Prostate**	Bladder	Max DVH	35 Gy(RBE) to 20 % volume	50
	Femoral Head Left	Max DVH	22.5 Gy(RBE) to 50 % volume	50
	Femoral Head Right	Max DVH	22.5 Gy(RBE) to 50 % volume	50
	Bowel	Max dose	25 Gy(RBE)	50
	Rectum	Max DVH	39.5 Gy(RBE) to 5 % volume	50
	Target (GTV)	Uniform dose, robust	40 Gy(RBE)	100
**H&N**	Eye left	Max DVH	25 Gy(RBE) to 1 % volume	100
	Eye right	Max DVH	25 Gy(RBE) to 1 % volume	100
	Mandibula	Max DVH	70 Gy(RBE) to 1 % volume	100
	Brainstem	Max DVH, robust	54 Gy(RBE) to 1 % volume	100
	Optic nerve left	Max DVH, robust	54 Gy(RBE) to 1 % volume	100
	Optic nerve right	Max DVH, robust	54 Gy(RBE) to 1 % volume	100
	Chiasm	Max DVH, robust	55 Gy(RBE) to 1 % volume	100
	Target (CTV)	Max DVH, robust	70 Gy(RBE) to 1 % volume	1000
	Target (CTV)	Min DVH, robust	69 Gy(RBE) to 99 % volume	1000

Table S2: Number of protons ($\times 10^6$) initially set and then actually assigned to the boosted spots after $D_{98\%}$ rescaling, when plans were boosted with the same number of protons to the selected spots.

**# boosted spots per beam →**		1	2	3	4	5
**Patient**	**Set # protons**	**# protons after D_{98} resc.**				
Prostate	200	202.9	202.9	202.8	203.2	203.3
	300	304.2	304.6	305.7	305.9	305.5
	400	406.6	407.2	408.4	408.6	410.8
	500	508.4	510.9	511.0	515.5	521.8
	600	612.4	612.9	617.6	623.8	632.8
	700	718.8	720.6	731.1	734.8	757.5
	800	824.8	827.8	846.1	865.5	877.6
H&N	200	197.4	197.2	196.6	197.1	196.2
	300	295.1	295.5	296.2	297.0	296.1
	400	393.9	394.0	395.2	397.0	395.4
	500	492.2	492.4	493.1	495.5	495.3
	600	587.7	599.2	595.6	598.8	598.8
	700	690.6	700.2	701.3	704.7	705.3
	800	793.1	810.2	811.6	814.8	818.1

Table S3: $D_{2\%}$ (Gy(RBE)), $L_{2\%}$ (keV/μm) and $LD_{2\%}$ (Gy) for the rectum contour for all the boosting cases evaluated, including $D_{98\%}$ rescaling, and relative differences (in brackets) to the plan before boosting, exhibiting initial values of $D_{2\%}$ = 40.4 Gy(RBE), $L_{2\%}$ = 6.8 keV/μ, $LD_{2\%}$ = 41.8 Gy. Plans were boosted with the same number of protons per selected spot.

# Protons per Boosted Spot ($\times 10^8$)	# Boosted Spots per Beam				
**$D_{2\%}$**	1	2	3	4	5
2	40.6 (0.5 %)	40.5 (0.3 %)	40.3 (-0.2 %)	40.7 (0.9 %)	40.7 (0.9 %)
3	40.7 (0.7 %)	40.4 (0.1 %)	40.7 (0.8 %)	41.3 (2.3 %)	41.3 (2.3 %)
4	40.8 (1.1 %)	40.9 (1.4 %)	41.2 (1.9 %)	41.2 (2.0 %)	42.0 (3.9 %)
5	40.6 (0.5 %)	41.3 (2.3 %)	41.3 (2.3 %)	41.4 (2.5 %)	43.2 (7.0 %)
6	40.5 (0.4 %)	41.0 (1.4 %)	41.5 (2.7 %)	43.1 (6.7 %)	42.6 (5.6 %)
7	41.2 (2.2 %)	41.3 (2.3 %)	42.0 (4.1 %)	42.5 (5.3 %)	44.6 (10.5 %)
8	41.3 (2.3 %)	41.9 (3.7 %)	42.0 (4.0 %)	43.9 (8.6 %)	43.6 (8.0 %)
**$L_{2\%}$**	1	2	3	4	5
2	7.1 (4.4 %)	7.0 (2.9 %)	6.7 (-1.5 %)	6.9 (1.5 %)	7.9 (16.2 %)
3	6.7 (-1.5 %)	6.9 (1.5 %)	7.0 (2.9 %)	7.2 (5.9 %)	7.5 (10.3 %)
4	6.9 (1.5 %)	7.3 (7.4 %)	7.3 (7.4 %)	7.2 (5.9 %)	7.1 (4.4 %)
5	6.9 (1.5 %)	7.4 (8.8 %)	7.7 (13.2 %)	7.6 (11.8 %)	7.4 (8.8 %)
6	7.5 (10.3 %)	7.1 (4.4 %)	7.8 (14.7 %)	7.5 (10.3 %)	7.3 (7.4 %)
7	7.7 (13.2 %)	6.9 (1.5 %)	6.9 (1.5 %)	7.3 (7.4 %)	7.3 (7.4 %)
8	7.3 (7.4 %)	7.2 (5.9 %)	7.4 (8.8 %)	7.4 (8.8 %)	7.5 (10.3 %)
**$LD_{2\%}$**	1	2	3	4	5
2	42.1 (0.7 %)	42.0 (0.5 %)	41.7 (-0.2 %)	42.2 (1.0 %)	42.2 (1.0 %)
3	42.1 (0.7 %)	41.7 (-0.2 %)	42.1 (0.7 %)	42.7 (2.2 %)	42.9 (2.6 %)
4	42.3 (1.2 %)	42.4 (1.4 %)	42.6 (1.9 %)	42.5 (1.7 %)	43.7 (4.5 %)
5	42.2 (1.0 %)	42.6 (1.9 %)	42.7 (2.2 %)	43.1 (3.1 %)	45.4 (8.6 %)
6	42.0 (0.5 %)	42.5 (1.7 %)	42.9 (2.6 %)	44.7 (6.9 %)	44.3 (6.0 %)
7	42.8 (2.4 %)	42.8 (2.4 %)	43.6 (4.3 %)	44.3 (6.0 %)	47.0 (12.4 %)
8	43.0 (2.9 %)	43.5 (4.1 %)	43.9 (5.0 %)	45.8 (9.6 %)	46.4 (11.0 %)

Table S4: $D_{2\%}$ (Gy(RBE)), $L_{2\%}$ (keV/μm) and $LD_{2\%}$ (Gy) for the brainstem contour for all the boosting cases evaluated, including $D_{98\%}$ rescaling, and relative differences (in brackets) to the plan before boosting, exhibiting initial values of $D_{2\%} = 62.3$ Gy(RBE), $L_{2\%} = 8.4$ keV/μm, $LD_{2\%} = 66.4$ Gy. Plans were boosted such with the reward function in use, with a fixed weight $w = 1$.

Steepness	# Boosted Spots per Beam				
**$D_{2\%}$**	1	2	3	4	5
0.0	62.2 (-0.1 %)	62.0 (-0.5 %)	61.8 (-0.8 %)	62.3 (-0.0 %)	61.5 (-1.3 %)
0.5	61.4 (-1.5 %)	61.2 (-1.8 %)	61.8 (-0.8 %)	62.0 (-0.5 %)	61.8 (-0.8 %)
1.0	61.1 (-1.9 %)	61.1 (-2.0 %)	61.5 (-1.4 %)	61.6 (-1.1 %)	61.9 (-0.6 %)
3.0	62.1 (-0.4 %)	61.7 (-1.0 %)	61.5 (-1.3 %)	61.8 (-0.9 %)	61.4 (-1.5 %)
5.0	61.8 (-0.8 %)	62.0 (-0.4 %)	61.6 (-1.1 %)	62.0 (-0.6 %)	61.3 (-1.7 %)
**$L_{2\%}$**	1	2	3	4	5
0.0	8.2 (-3.4 %)	8.2 (-2.5 %)	8.4 (-0.5 %)	8.1 (-4.4 %)	8.4 (-0.1 %)
0.5	8.4 (-0.5 %)	9.1 (7.6 %)	9.2 (8.5 %)	9.7 (15.3 %)	9.5 (12.4 %)
1.0	8.7 (2.4 %)	8.7 (2.7 %)	8.8 (4.2 %)	9.6 (13.4 %)	9.2 (8.8 %)
3.0	8.5 (0.7 %)	8.6 (1.4 %)	8.6 (2.1 %)	8.8 (3.7 %)	8.8 (4.5 %)
5.0	8.5 (0.5 %)	8.4 (-0.3 %)	8.4 (-0.7 %)	8.6 (2.1 %)	8.5 (0.7 %)
**$LD_{2\%}$**	1	2	3	4	5
0.0	66.2 (-0.3 %)	66.2 (-0.4 %)	65.8 (-1.0 %)	66.4 (-0.1 %)	65.9 (-0.8 %)
0.5	65.9 (-0.8 %)	65.6 (-1.2 %)	66.7 (0.4 %)	67.4 (1.5 %)	66.3 (-0.1 %)
1.0	65.2 (-1.8 %)	65.5 (-1.3 %)	66.7 (0.4 %)	66.8 (0.6 %)	66.6 (0.2 %)
3.0	66.0 (-0.7 %)	66.6 (0.3 %)	66.6 (0.2 %)	67.0 (0.9 %)	66.1 (-0.5 %)
5.0	66.4 (0.0 %)	66.3 (-0.1 %)	66.2 (-0.3 %)	66.6 (0.3 %)	65.5 (-1.3 %)

Table S5: $D_{2\%}$ (Gy(RBE)), $L_{2\%}$ (keV/μm) and $LD_{2\%}$ (Gy) for the rectum contour for all the boosting cases evaluated, including $D_{98\%}$ rescaling, and relative differences (in brackets) to the plan before boosting, exhibiting initial values of $D_{2\%}$ = 40.4 Gy(RBE), $L_{2\%}$ = 6.8 keV/μ, $LD_{2\%}$ = 41.8 Gy. Plans were boosted with the reward function in use, with a fixed weight $w = 1$.

Steepness	# Boosted Spots per Beam				
**$D_{2\%}$**	1	2	3	4	5
0.0	40.7 (0.7 %)	40.5 (0.2 %)	40.5 (0.2 %)	40.5 (0.2 %)	40.9 (1.2 %)
0.5	41.3 (2.2 %)	41.8 (3.5 %)	44.4 (9.9 %)	44.2 (9.4 %)	45.0 (11.4 %)
1.0	41.2 (2.0 %)	41.9 (3.7 %)	41.8 (3.5 %)	41.9 (3.7 %)	43.3 (7.2 %)
3.0	40.9 (1.2 %)	41.4 (2.5 %)	40.9 (1.2 %)	41.6 (3.0 %)	42.3 (4.7 %)
5.0	40.7 (0.7 %)	41.0 (1.5 %)	41.2 (2.0 %)	41.3 (2.2 %)	41.3 (2.2 %)
**$L_{2\%}$**	1	2	3	4	5
0.0	6.9 (1.5 %)	7.0 (2.9 %)	6.9 (1.5 %)	6.9 (1.5 %)	7.2 (5.9 %)
0.5	7.6 (11.8 %)	7.2 (5.9 %)	7.2 (5.9 %)	7.1 (4.4 %)	7.4 (8.8 %)
1.0	6.9 (1.5 %)	7.8 (14.7 %)	7.1 (4.4 %)	7.1 (4.4 %)	7.4 (8.8 %)
3.0	7.4 (8.8 %)	7.6 (11.8 %)	7.0 (2.9 %)	6.9 (1.5 %)	7.3 (7.4 %)
5.0	6.8 (0.0 %)	7.2 (5.9 %)	7.7 (13.2 %)	6.8 (0.0 %)	7.3 (7.4 %)
**$LD_{2\%}$**	1	2	3	4	5
0.0	42.1 (0.7 %)	41.9 (0.2 %)	42.0 (0.5 %)	41.9 (0.2 %)	42.3 (1.2 %)
0.5	42.9 (2.6 %)	43.3 (3.6 %)	46.8 (11.9 %)	46.7 (11.7 %)	47.8 (14.4 %)
1.0	42.8 (2.4 %)	43.8 (4.8 %)	43.3 (3.6 %)	43.8 (4.8 %)	45.4 (8.6 %)
3.0	42.3 (1.2 %)	43.0 (2.9 %)	42.2 (1.0 %)	43.0 (2.9 %)	44.2 (5.7 %)
5.0	42.2 (1.0 %)	42.4 (1.4 %)	42.6 (1.9 %)	42.6 (1.9 %)	42.8 (2.4 %)

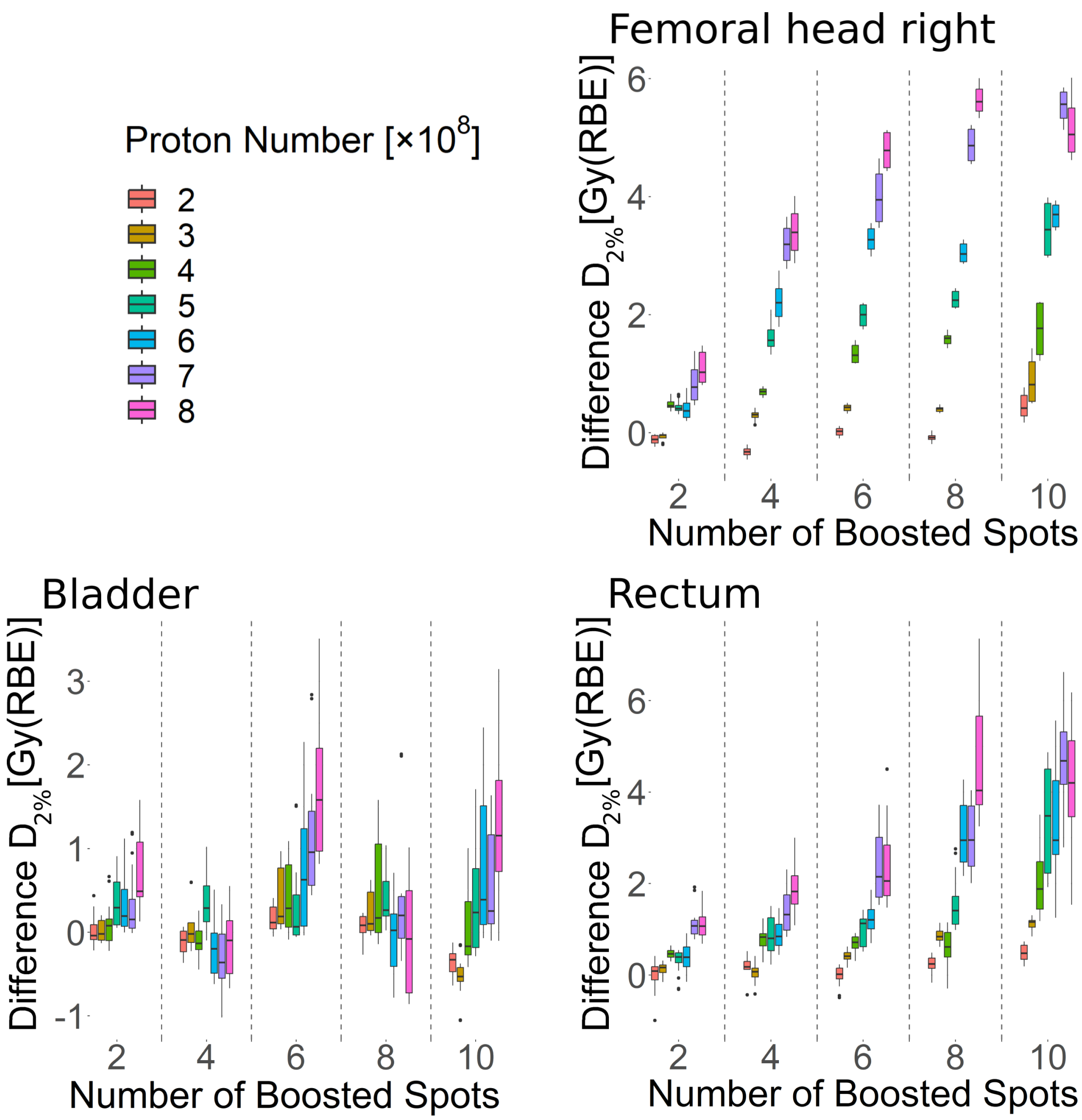

Fig. S2: Robustness evaluation reporting the absolute differences in the considered metrics between respective scenarios in the boosted and initial plans. Results related to OOIs belonging to the prostate patient are provided. Spots were boosted with the same number of protons, reported in the legend. $D_{98\%}$ rescaling was applied.

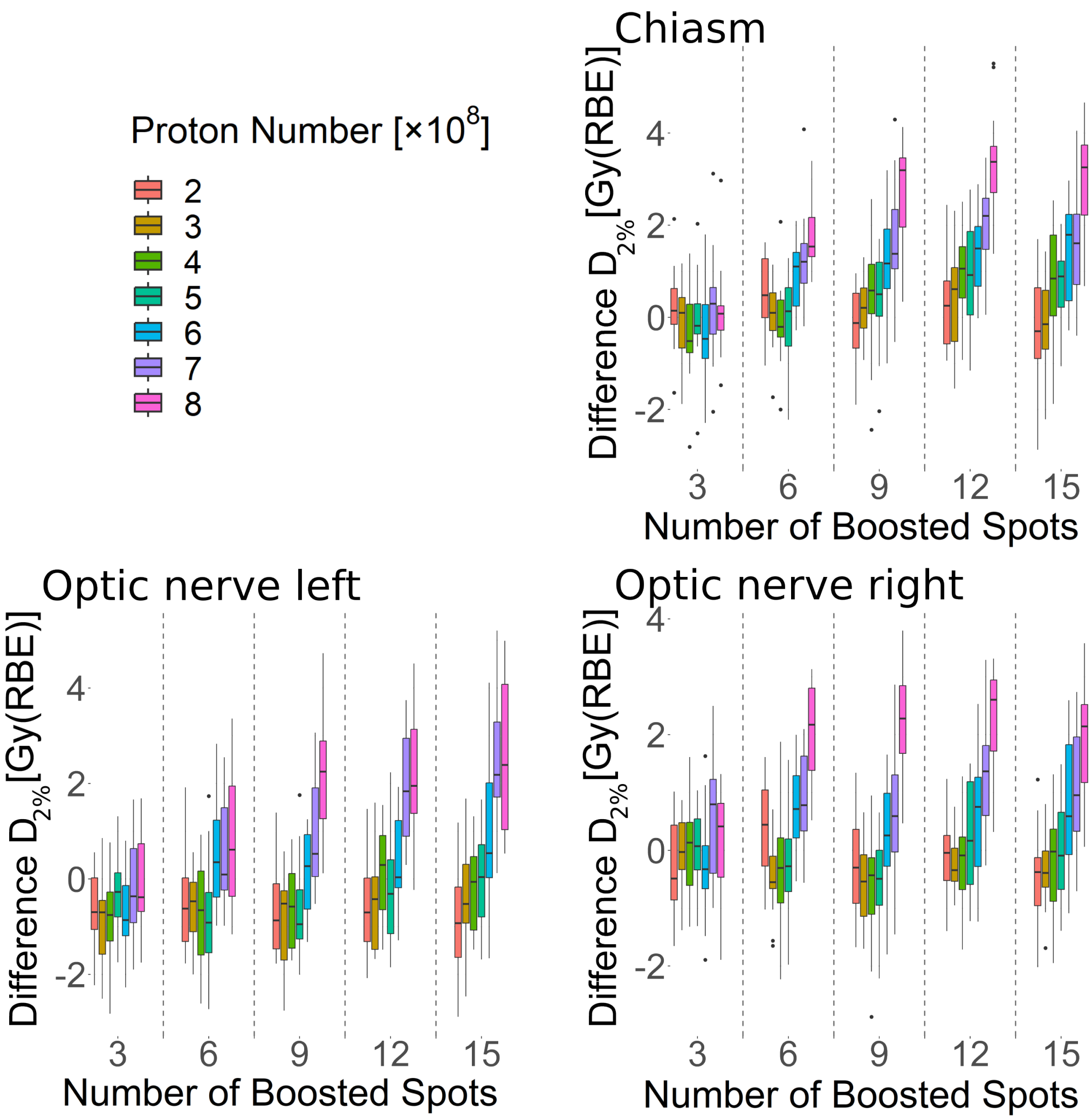

Fig. S3: Robustness evaluation reporting the absolute differences in the considered metrics between respective scenarios in the boosted and initial plans. Results related to OOIs belonging to the H&N patient are provided. Spots were boosted with the same number of protons, reported in the legend. $D_{98\%}$ rescaling was applied.

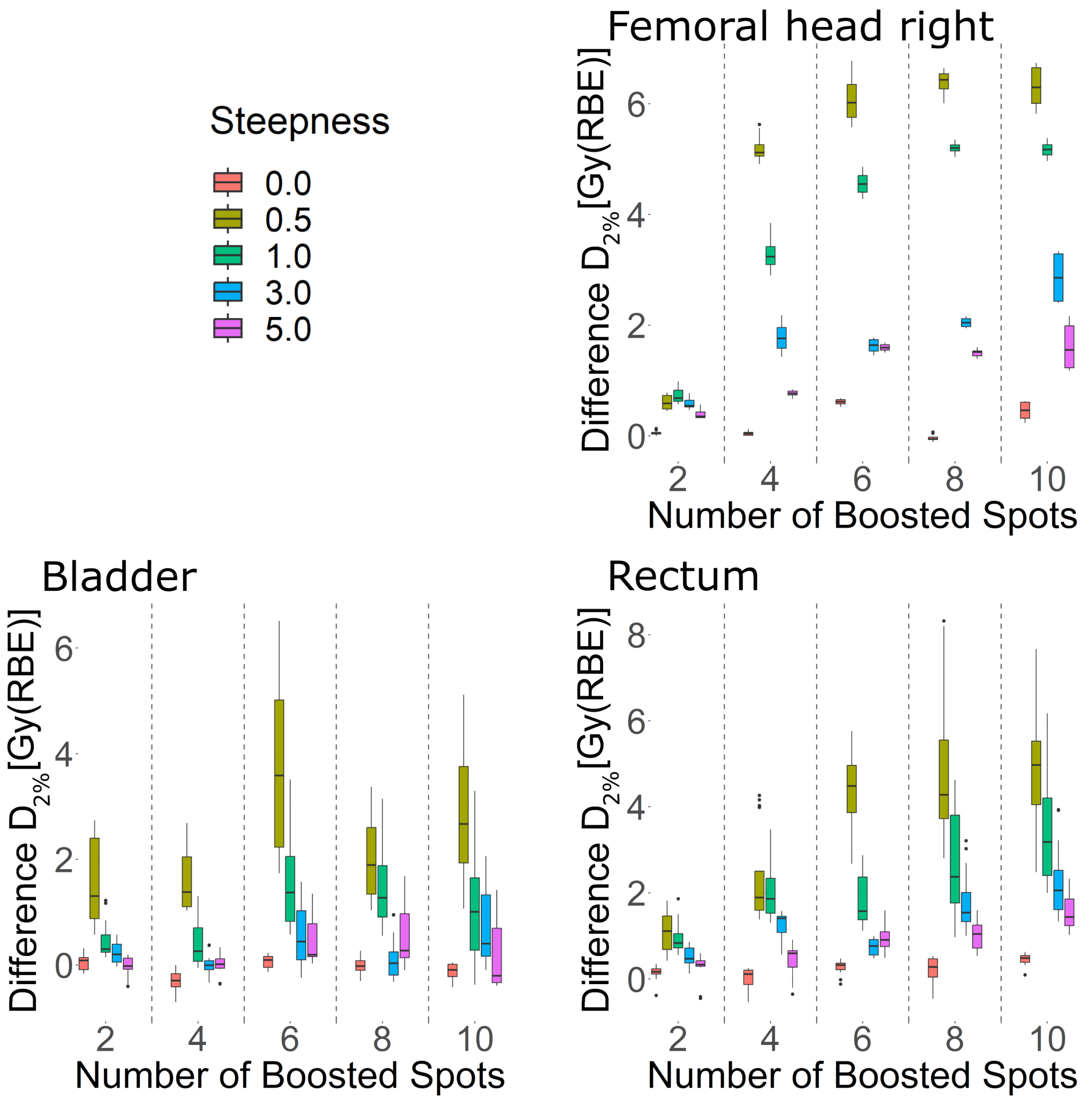

Fig. S4: Robustness evaluation reporting the absolute differences in the considered metrics between respective scenarios in the boosted and initial plans. Results related to OOIs belonging to the prostate patient are provided. Spots were boosted with the reward function in use. $D_{98\%}$ rescaling was applied.

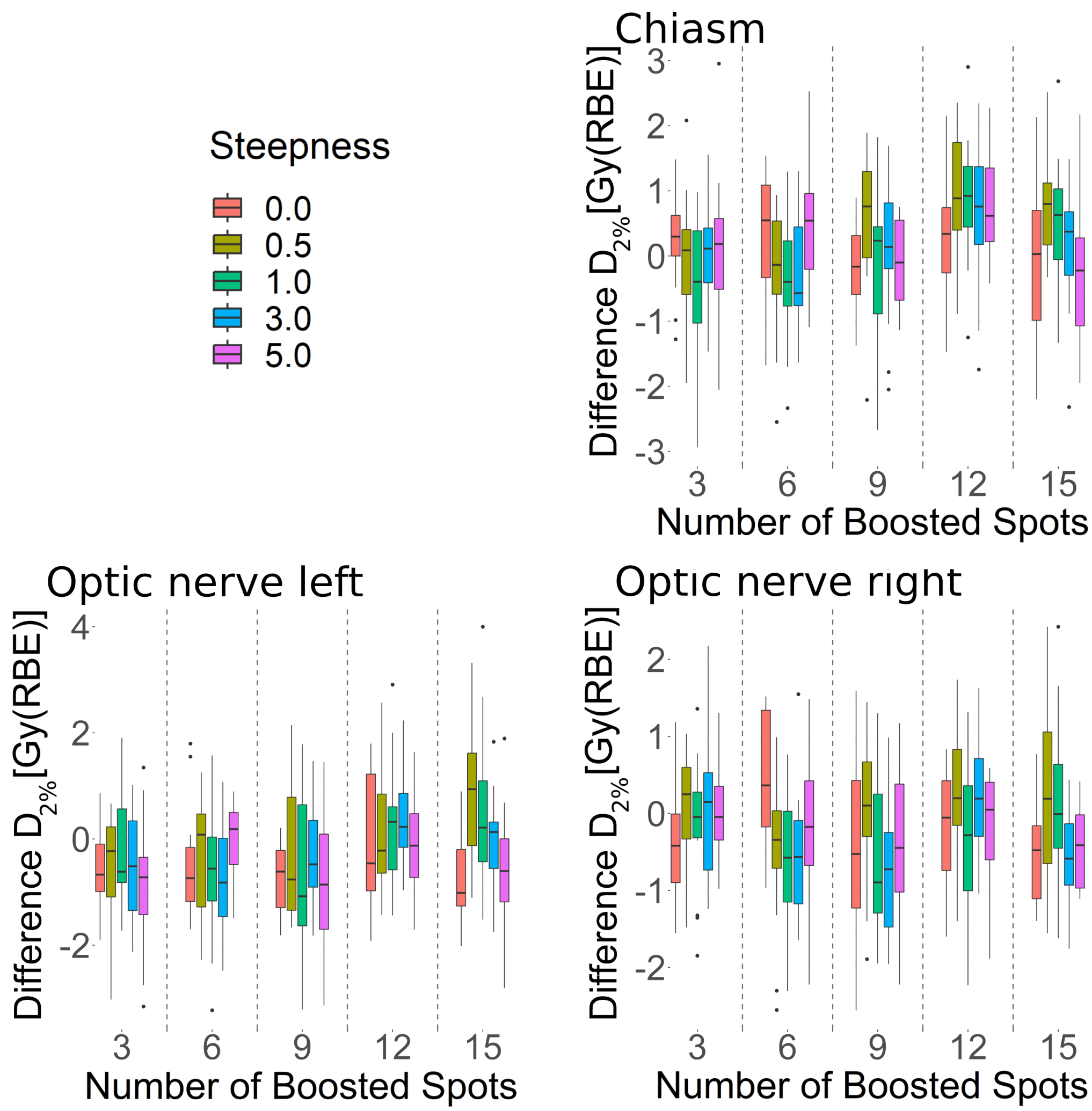

Fig. S5: Robustness evaluation reporting the absolute differences in the considered metrics between respective scenarios in the boosted and initial plans. Results related to OOIs belonging to the H&N patient are provided. Spots were boosted with the reward function in use. $D_{98\%}$ rescaling was applied.
